# Sequentially mediated effects of weight-related self-stigma and psychological distress in the association between perceived weight stigma and food addiction among Taiwanese university students: A cross-sectional study

**DOI:** 10.1186/s40337-022-00701-y

**Published:** 2022-11-21

**Authors:** Po-Ching Huang, Chiu-Hsiang Lee, Mark D. Griffiths, Kerry S. O’Brien, Yi-Ching Lin, Wan Ying Gan, Wai Chuen Poon, Ching-Hsia Hung, Kuo-Hsin Lee, Chung-Ying Lin

**Affiliations:** 1 Institute of Allied Health Sciences, College of Medicine, National Cheng Kung University, No. 1, University Rd., East Dist., Tainan, 701401 Taiwan; 2 Department of Nursing, Chung Shan Medical University, No. 110, Sec. 1, Jianguo N. Rd., South Dist., Taichung, 402306 Taiwan; 3 Department of Nursing, Chung Shan Medical University Hospital, No. 110, Sec. 1, Jianguo N. Rd., South Dist., Taichung, 402306 Taiwan; 4 International Gaming Research Unit, Psychology Department, Nottingham Trent University, 50 Shakespeare Street, Nottingham, NG1 4FQ UK; 5 School of Social Sciences, Faculty of Arts, Monash University, 20 Chancellors Walk, Clayton, VIC 3800 Australia; 6 Department of Early Childhood and Family Education, National Taipei University of Education, No. 134, Sec. 2, Heping E. Rd., Da’an Dist., Taipei, 106320 Taiwan; 7 Department of Nutrition, Faculty of Medicine and Health Sciences, Universiti Putra Malaysia, 43400 Serdang, Selangor Malaysia; 8 Sunway University Business School, Sunway University, Selangor Darul Ehsan, No. 5, Jalan Universiti, 47500 Bandar Sunway, Malaysia; 9 Department of Physical Therapy, College of Medicine, National Cheng Kung University, No. 1, Daxue Rd., East Dist., Tainan, 701401 Taiwan; 10 Department of Emergency Medicine, E-Da Hospital, I-Shou University, No.1, Yida Rd, Yanchao District, Kaohsiung City, 824 Taiwan; 11 School of Medicine, I-Shou University, No. 8, Yi-Da Road, Jiao-Su Village, Yan-Chao District, Kaohsiung City, 824 Taiwan; 12 Department of Occupational Therapy, College of Medicine, National Cheng Kung University, No. 1, University Rd., East Dist., Tainan, 701401 Taiwan; 13 Department of Public Health, College of Medicine, National Cheng Kung University, No. 1, University Rd., East Dist., Tainan, 701401 Taiwan; 14 Biostatistics Consulting Center, National Cheng Kung University Hospital, College of Medicine, National Cheng Kung University, No. 1, University Rd., East Dist., Tainan, 701401 Taiwan

**Keywords:** Food addiction, Mediation model, Psychological distress, Weight bias, Weight stigma

## Abstract

**Background:**

Weight-related stigma has negative physiological and psychological impacts on individuals’ quality of life. Stigmatized individuals may experience higher psychological distress and therefore increase the potential risk to develop obesity and/or food addiction. The present study examined the associations and mediated effect between perceived weight stigma, weight-related self-stigma, and psychological distress in explaining food addiction among Taiwanese university students.

**Methods:**

All participants (n = 968) completed an online survey which included the Perceived Weight Stigma Questionnaire, Weight Self-Stigma Questionnaire, Depression, Anxiety, Stress Scale-21, and Yale Food Addiction Scale Version 2.

**Results:**

After controlling for demographic variables, significant associations were found in the paths from (1) perceived weight stigma to weight-related self-stigma ($\beta$ = 0.23), psychological distress ($\beta$ = 0.35), and food addiction ($\beta$ = 0.23); (2) weight-related self-stigma to psychological distress ($\beta$ = 0.52) and food addiction ($\beta$ = 0.59); and (3) psychological distress to food addiction ($\beta$ = 0.59) (all *p-*values < 0.001). The mediation model showed the sequential mediated effect of weight-related self-stigma and psychological distress in the association between perceived weight stigma and food addiction.

**Conclusions:**

The results provide novel insights that weight-related self-stigma and psychological distress sequentially mediated the relationship between perceived weight stigma and food addiction among Taiwanese university students. The findings of the present study could be implemented into interventions that aim to reduce food addiction derived from weight-related stigma. Future studies should consider group analysis to consider confounding factors or other populations to provide more evidence regarding the mechanism of weight-related stigma.

## Plain English Summary

Weight-related stigma could cause a negative influence on both physiological and psychological status of stigmatized individuals. The possibility of developing food addiction may exacerbate obesity and further worsen weight-related stigma. The present study investigated the association between perceived weight stigma, weight-related self-stigma, and psychological distress, and food addiction among university students. The additional mediated effect was determined as well. All respondents (n = 968) completed an online survey including the Perceived Weight Stigma Questionnaire, Weight Self-Stigma Questionnaire, Depression, Anxiety, Stress Scale-21, and Yale Food Addiction Scale Version 2. Significant associations were found between all the investigated variables with the control of demographic variables. The mediation models further revealed the sequential mediated effect of weight-related self-stigma and psychological distress in the relationship of perceived weight stigma and food addiction. Accordingly, the present study provides the novel insights of the mediated role of weight-related self-stigma and psychological distress regarding the development of food addition in the mechanism of weight-related stigma. The current findings could be implemented into the development of interventions targeted on reducing the food addiction derived from weight-related stigma.

## Introduction

Weight-related stigma is a social problem that impacts on both physiological and psychological health [1]. Individuals who suffer from weight stigma may experience physiological responses including changes in cortisol and oxidative stress levels [2], alongside the possibility of weight gain [3], obesity [3] and increased diabetes risk [4], as well as the psychological impacts such as depression, anxiety, low self-esteem [5] and associated eating disturbance [5]. Consequently, the unpleasant social experience and undervalued burden end up negatively affecting the quality of life of stigmatized individuals [6].

Weight-related stigma is defined as the prejudice, negative stereotyping, and discrimination derived from the devaluation and denigration experienced by individuals who perceive themselves as having excessive weight [7]. The perception of individuals plays a crucial role on the formation of weight stigmatization [7, 8], and is more important than the objective realities to the stigmatized individuals [8, 9]. Ratcliffe and Ellison (2015) proposed a model regarding the impact of external devaluation on internal self-evaluation, indicating that the undesired external judgment such as negative stereotypes (e.g., being referred to as “lazy” or “lack of self-discipline”) or hostile behavior (e.g., being teased or bullied about their body shape) reduces self-esteem [5] and increases the vulnerability of individuals who are obese to experiencing internalized weight stigma. In addition, the greatest weight stigmatization has frequently been reported to be by relatives or close friends [7, 10, 11] and one study supported the notion that the closer the relationship to the source of weight stigma, the stronger the impact on weight stigma internalization and weight self-stigma [11].

Eating food acts as a comfort-seeking behavior in response to perceived stress, and endogenous negative psychological distress is commonly seen among stigmatized individuals [12]. Weight-related stigma has been identified as a psychosocial stressor [13] because unfriendly external devaluation and internal self-judgement [8] cause the severe psychological burden to stigmatized individuals. Stress appraisal [14] not only elicits negative emotional responses such as depression and anxiety [5, 13], but can result in excessive eating being used as a coping strategy [12, 14]. Chronic stress has been reported to be one of the potential mechanisms which alters neural responses and regulates behavior, causing individuals to develop eating disorders such as binge eating and/or emotional eating [12] because food consumption acts as an intrinsic rewarding and reinforcing behavior which sufficiently activates the brain reward system [15, 16].

The cyclic obesity/weight-based stigma (COBWEBS) model [7] illustrates the vicious cycle between weight stigma and obesity. It proposes that the stress generated from weight stigma provokes the eating disorder and increases food intake. The consequential weight gain and obesity could worsen the weight stigma towards stigmatized individuals and continues the loop. Additionally, specific mental health disorders, such as depression, may be associated with the development of food addiction [17]. Food addiction refers to addictive-like eating behavior [18, 19] in which individuals are unable to self-regulate their eating behavior and lose control of their food intake [20]. Although the concept of food addiction is still debated [19, 21], there are studies reporting the association of food addiction with obesity and increased risk of cardiovascular [22] and/or metabolic [4] diseases. Studies have shown that advanced emotional dysregulation (including low self-esteem, depression, and negative emotions) are found among individuals who identify as having a food addiction [17, 21]. The results of previous studies suggest that psychological distress induced by weight-related stigma predict the development of psychopathological food addiction [17, 21]. Moreover, other studies have postulated that it is the fear of being stigmatized from others rather than weight-related self-devaluation that helps explain the development of food addiction [23]. However, the relationships between these factors remains unclear.

It has also been reported that university students as a specific cohort may have a potential risk to experiencing weight stigma. Students at this stage of their life may experience independent living for the first time, especially Taiwanese youth. More specifically, most Taiwanese live with their parents and are under their supervision until they begin university. Without parental supervision, university students may not have boundaries and/or protections in place, and may develop potentially addictive behaviors (to such things as internet use or food intake) [24]. In addition, the continuous weight gain has been reported among university students in several countries [25]. The vulnerability to the predominant social model and representation experienced by university students [26] may increase the misperception of being overweight (i.e., overestimated weight status) [27–29] and body dissatisfaction (i.e., the negative subjective evaluation of weight and body shape) [28, 30]. The unwanted weight label (i.e., being labelled due to weight or body shape) could have negative consequences, such as increased internalized weight stigma and poor perceived health [31]. Once individuals perceive weight-related stigma, the vulnerability experienced during this particular period, along with the awareness regarding weight and body shape, increases the possibility of weight stigmatization among university students.

In the present study, weight stigma derived from others is referred to as perceived weight stigma (e.g., *“being stared at by the public”*) and its internalization is identified as weight-related self-stigma (e.g., *“I’m useless because of my weight”*) [32]. Therefore, the purpose of the present study was to investigate the mediated effect of perceived weight stigma, weight-related self-stigma, and psychological distress on food addiction among university students. It was hypothesized that weight-related self-stigma and psychological distress would act as mediators in the relationship between perceived weight stigma and food addiction.

## Methods

### Study design and participants

The study was a cross-sectional online survey hosted on the *Google Forms* platform from August, 2021 to April, 2022. The target participants were university students who were studying at any university in Taiwan. The inclusion criteria of the participants were (i) being aged 20 years or above; (ii) residing in Taiwan during the survey period; and (iii) possessing a Wi-Fi-enabled device that could access the online survey on *Google Forms*. Individuals who were international students or who did not report their age were excluded from participation in the study. However, for participants who did not provide informed consent to participate, they were directed to the end of the survey without completing any of the survey items. For those who agreed to participate in the study, they were requested to click an ‘*agree*’ icon to indicate their informed consent and willingness to take part. Then, they were led to the survey questions and all the survey questions were presented in Chinese (please see *Measures* section below for details). The survey distribution included use of the snowball sampling method. More specifically, the authors invited their colleagues teaching at universities to disseminate the survey information (via weblink or QR code) to their students. Participants were also encouraged to share the survey information with their university friends.

Before the participants selected ‘*agree*’ or ‘*disagree*’ to participate in the present study, they were well informed about the study purpose and their participation rights in the first page of *Google Forms*. Additionally, the entire study process adhered to and complied with the Declaration of Helsinki. Their informed consent for participation was indicated by pressing the ‘*agree*’ icon before entering the survey. Each participant received a financial incentive of 100 New Taiwan Dollars (approximately $3.3 US) if they completed all the survey questions. The present study was approved by the Institutional Review Board in the Chi Mei Medical Center (IRB Serial No.: 11007-006) and the Human Research Ethics Committee in the National Cheng Kung University (Approval No.: NCKU HREC-E-109-551-2).

### Measures

#### Perceived Weight Stigma Questionnaire (PWSQ)

The PWSQ [33, 34] contains 10 items (e.g., “*You are treated with less respect than others*”) utilizing dichotomous scoring (0 = no; 1 = yes). The scores (ranging from 0 to 10) are summed and a higher score indicates a higher level of perceived weight stigma. Previous research has reported a good internal consistency of the Chinese PWSQ [35]. The internal consistency of the PWSQ in the present study was excellent (α = 0.92).

#### Weight Self-Stigma Questionnaire (WSSQ)

The WSSQ [33, 34, 36] contains 12 items (e.g., “*I caused my weight problems*”) rated on a five-point Likert scale (1 = completely disagree; 5 = completely agree). The scores (ranging from 12 to 60) are summed and a higher score indicates a greater level of self-stigma derived from individuals’ weight or body shape. Robust psychometric properties (including construct validity, concurrent validity, test–retest reliability, and internal consistency) of the Chinese WSSQ have been found in previous research [30, 37]. The Chinese WSSQ has also been validated using an adult Taiwanese sample [35]. The internal consistency of the WSSQ in the present study was excellent (α = 0.94).

#### Depression, Anxiety, Stress Scale-21 (DASS-21)

The DASS-21 [38] contains 21 items (e.g., “*I found it difficult to relax*”) rated on a four-point Likert scale (0 = Did not apply to me at all; 3 = Applied to me very much or most of the time). The scores (ranging from 0 to 63) are summed and a higher score indicates a higher level of psychological distress. Robust psychometric properties (including construct validity, concurrent validity, test–retest reliability, and internal consistency) of the Chinese DASS-21 have been found in previous research [39, 40]. The internal consistency of the DASS-21 in the present study was excellent (α = 0.97).

#### Yale Food Addiction Scale Version 2 (YFAS 2.0)

The YFAS 2.0 [41] contains 35 items (e.g., “*In the past 12 months, when I started to eat certain foods, I ate much more than planned*”) rated on an eight-point scale (0 = never; 7 = everyday). The YFAS 2.0 adopts a unique scoring method [41] wherein 35 items belonging to 11 symptoms were converted into a 0–1 dichotomous scale (0 indicates *non-endorsed*; 1 indicates *endorsed*). Total scores range from 0 to 11 and a higher score indicates a greater risk of food addiction and a cut-off value $\ge$ 2 indicates at-risk/food addiction [42]. Robust psychometric properties (including construct validity, concurrent validity, test–retest reliability, and internal consistency) of the Chinese YFAS 2.0 have been found in previous research [43, 44]. The internal consistency of the YFAS 2.0 in the present study was excellent (α = 0.99).

#### Background information

The background information section asked questions regarding the participants’ height (in cm), weight (in kg), age (in years), and gender (male or female). The information concerning height and weight was later used to calculate their body mass index (BMI) with the unit of kg/m^2^.

### Statistical analysis

The participants’ background information and their scores on the measures were summarized using descriptive statistics. Then, the studied variables in the present study were computed for their associations using the Pearson correlation. Hierarchical regression models were subsequently carried out to examine how the PWSQ, WSSQ, and DASS-21 scores explained the variance in YFAS 2.0 score. In the hierarchical regression models, there were three models with YFAS 2.0 score being the dependent variable. In Model 1, age, gender, and BMI were entered; in Model 2, the PWSQ and WSSQ scores were additionally entered; in Model 3, the DASS-21 score was further entered. Finally, a sequential mediation model was constructed using Model 6 in Hayes’ Process Macro [45]. In the sequential mediation model, YFAS 2.0 score was the dependent variable, PWSQ score was the independent variable, WSSQ and DASS-21 scores were the sequential mediators with age, gender, and BMI being controlled for. A total of 5000 bootstrapping resamples were used in the sequential mediation model. A significant mediated effect (or indirect effect) was supported when the 95% bootstrapping confidence interval (CI) do not cover 0 [46]. All the statistical analyses were performed using IBM SPSS 20.0 (IBM, Corp., NY: Armonk).

## Results

Table 1 presents the characteristics of the present sample (n = 968). In sum, the sample was relatively young (mean age = 23.7 years; SD = 4.3) with more females than males (59.5% females). The average BMI of the present sample was 22.4 kg/m^2^ (SD = 3.6). The mean scores of the measures were 2.1 out of 10 (PWSQ), 31.7 out of 60 (WSSQ), 18.1 out of 63 (DASS-21), and 2.26 out of 11 (YFAS 2.0).

**Table 1:** Characteristics of the university students (N = 968)

	Mean (SD)
Age (year)	23.7 (4.3)
Gender (male)^a^	392 (40.5)
Height (cm)	165.9 (8.1)
Weight (kg)	61.8 (12.6)
Body mass index (kg/m^2^)	22.4 (3.6)
PWSQ score (possible score range: 0–10)	2.1 (3.1)
WSSQ score (possible score range: 12–60)	31.7 (10.3)
DASS-21 score (possible score range: 21–63)	18.1 (15.3)
YFAS 2.0 score (possible score range: 0–11)	2.26 (3.0)

^a^Reported in n (%)

*PWSQ* Perceived Weight Stigma Questionnaire (10 items); *WSSQ* Weight Self-Stigma Questionnaire (12 items); *DASS-21* Depression, Anxiety, Stress Scale-21 (21 items), *YFAS 2.0* Yale Food Addiction Scale Version 2 (35 items)

Table 2 reports the correlations between the studied variables. In sum, BMI, PWSQ score, WSSQ score, DASS-21 score, and YFAS 2.0 score were significantly and positively correlated with each other (*p*-values < 0.001). More specifically, YFAS 2.0 score was strongly correlated with WSSQ and DASS-21 scores (*r* = 0.56 and 0.54); WSSQ score and DASS-21 score were also strongly correlated (*r* = 0.52).

**Table 2:** Correlations between the studied variables among the university students (N = 968)

				*r* (*p*-value)			
	Age	Gender	BMI	PWSQ	WSSQ	DASS-21	YFAS 2.0
Age	–						
Gender	− 0.03 (0.30)	–					
BMI	0.09 (0.01)	0.30 (< 0.001)	–				
PWSQ	0.04 (0.23)	0.26 (< 0.001)	0.20 (< 0.001)	–			
WSSQ	0.08 (0.01)	0.01 (0.71)	0.36 (< 0.001)	0.23 (< 0.001)	–		
DASS-21	0.09 (0.003)	0.08 (0.01)	0.14 (< 0.001)	0.35 (< 0.001)	0.52 (< 0.001)	–	
YFAS 2.0	0.07 (0.04)	0.05 (0.13)	0.17 (< 0.001)	0.20 (< 0.001)	0.56 (< 0.001)	0.54 (< 0.001)	–

Cronbach’s alpha = 0.91 (YFAS 2.0); 0.92 (PWSQ); 0.94 (WSSQ); 0.97 (DASS-21)

*PWSQ* Perceived Weight Stigma Questionnaire (10 items); *WSSQ* Weight Self-Stigma Questionnaire (12 items); *DASS-21* Depression, Anxiety, Stress Scale-21 (21 items); *YFAS 2.0* Yale Food Addiction Scale Version 2 (35 items)

Table 3 reports the results of the hierarchical regression models. BMI was a significant factor explaining YFAS 2.0 score in Model 1 (standardized coefficient [β] = 0.17; *p* < 0.001). However, the significant effects changed from positive to negative and nonsignificant when PWSQ and WSSQ scores were entered in the regression model (i.e., Model 2; β =  − 0.05; *p* = 0.07). The significant effects remained nonsignificant when DASS-21 score was additionally entered in the regression model (i.e., Model 3; β =  − 0.02; *p* = 0.51). PWSQ and WSSQ scores were significant factors explaining YFAS 2.0 score in Model 2 (β = 0.07 and 0.56, respectively; *p* = 0.02 and < 0.001). However, PWSQ score became nonsignificant when DASS-21 score was additionally entered (i.e., Model 3; β =  − 0.02; *p* = 0.48), while WSSQ score remained significant (β = 0.39; *p* < 0.001). DASS-21 was a significant factor explaining YFAS 2.0 score in Model 3 (β = 0.35; *p* < 0.001).

**Table 3:** Hierarchical regression models investigating the relationships between weight stigma, psychological distress, and food addiction (N = 968)

	Dependent variable: YFAS 2.0 Coeff. (SE)/Stand. Coeff. (*p*-value)		
	Model 1	Model 2	Model 3
Age	0.04 (0.02)/ 0.05 (0.10)	0.02 (0.02)/ 0.03 (0.35)	0.004 (0.02)/ 0.01 (0.80)
Gender (Ref: female)	0.005 (0.20)/ 0.001 (0.98)	0.26 (0.18)/ 0.04 (0.15)	0.17 (0.17)/ 0.03 (0.30)
Body mass index	0.14 (0.03)/ 0.17 (< 0.001)	− 0.05 (0.03)/ − 0.05 (0.07)	− 0.02 (0.02)/ − 0.02 (0.51)
PWSQ		0.07 (0.03)/ 0.07 (0.02)	− 0.02 (0.03)/ − 0.02 (0.48)
WSSQ		0.16 (0.009)/ 0.56 (< 0.001)	0.11 (0.01)/ 0.39 (< 0.001)
DASS-21			0.07 (0.01)/ 0.35 (< 0.001)
F-value (*p*-value)	10.97 (< 0.001)	89.29 (< 0.001)	105.24 (< 0.001)
R^2^ (adjusted R^2^)	0.03 (0.03)	0.32 (0.31)	0.40 (0.39)

*PWSQ* Perceived Weight Stigma Questionnaire (10 items); *WSSQ* Weight Self-Stigma Questionnaire (12 items); *DASS-21* Depression, Anxiety, Stress Scale-21 (21 items); *YFAS 2.0* Yale Food Addiction Scale Version 2 (35 items)

**Fig. 1:**
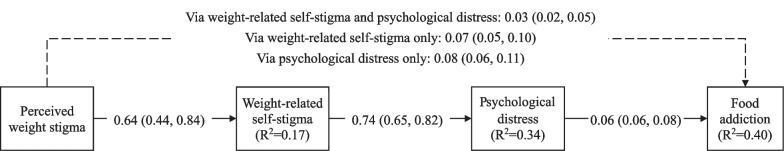
Mediated effects of psychological distress in the association between weight-related self-stigma and food addiction. Age, gender, and body mass index were controlled in the model (N = 968). Hayes’ Process Model 6 was used with 5000 bootstrapping samples. Dashed line indicates indirect effects; solid lines indicate direct effects. Coefficients reported using unstandardized coefficients with 95% confidence interval in parentheses.

The mediation model (Fig. 1) echoed the findings of the hierarchical regression models. PWSQ, WSSQ, and DASS-21 were significant factors explaining YFAS 2.0 score when age, gender, and BMI were controlled for. The mediated effects of WSSQ and DASS-21 scores in the association between PWSQ and YFAS 2.0 scores were supported. More specifically, the unstandardized coefficient (95% bootstrapping CI) was 0.03 (0.02, 0.05) for indirect effect via WSSQ and DASS-21 scores sequentially; and 0.07 (0.05, 0.10) for indirect effect via WSSQ score only; and the unstandardized coefficient (95% bootstrapping CI) was 0.08 (0.06, 0.11) for indirect effect via DASS-21 score only.

## Discussion

The present study investigated the mediation effect of weight-related self-stigma and psychological distress between perceived weight stigma and food addiction among Taiwanese university students. The results showed that perceived weight stigma, weight-related self-stigma, and psychological distress significantly explained food addiction, respectively. Moreover, weight-related self-stigma and psychological distress acted independently as significant mediators in the association between perceived weight stigma and food addiction. An additional sequential effect via weight-related self-stigma and psychological distress mediated the relationship between perceived weight stigma and food addiction. The findings of the present study support the proposed COBWEBS model [7] that the involvement of psychological distress affecting the development of food addiction was further demonstrated in the present study. Additionally, the findings support the notion that stress derived from obesity and weight-related stigma may elicit psychological distress to provoke the eating disorder and the individuals may be at increased risk of developing food addiction.

The relationship between perceived weight stigma and weight-related self-stigma has been reported in the literature [4, 47–49]. Similarly, the relationship between weight-related self-stigma and psychological distress has also been documented [49–51]. The present findings corroborate with the aforementioned relationships among Taiwanese university students. The relationships can be explained by the developed mechanism of weight-related stigma. That is, experienced weight stigma (i.e., prejudice or discrimination from others that was actually received by stigmatized individuals) elicited perceived weight stigma [48]. Once the individuals accepted the negative attributes regarding their weight, the stigma is internalized and initiates the process of weight-related self-stigmatization [8, 49, 50]. Additionally, the association between perceived weight stigma and psychological distress has also been investigated in previous studies [35, 49]. One study further reported that in the relationship between perceived weight stigma and psychological distress, the mediated effect of weight-related self-stigma was only observed among non-obese stigmatized individuals but not among those who were obese [49]. In other words, the psychological distress observed among individuals who are obese may be directly generated via perceived weight stigma [49] despite a higher level of weight-related self-stigma observed among individuals who are obese [50]. Therefore, future study is needed, focusing on the advanced consequences derived from weight-related self-stigma, such as eating disorders or decreased physical activity level, and should adopt group analysis according to individuals’ weight status, to better recognize the underlying mechanisms.

Both weight-related self-stigma and psychological distress acted as the mediators in the association between perceived weight stigma and food addiction. Several studies have supported the association between weight-related self-stigma and food addiction [51, 52]. However, more studies have focused on eating disorders (i.e., binge eating and/or emotional eating) rather than food addiction [5, 16, 23, 51, 52] because the growing evidence supports the notion of food addiction as a brain-derived mental health disorder but not the cause of obesity [16, 18]. To the best of the present authors’ knowledge, only one previous study [53] has addressed the involvement of perceived weight stigma in explaining eating disorders and no previous study has examined how perceived weight stigma explains food addiction. Therefore, the present findings extend the association between perceived weight stigma and eating disorder to perceived weight stigma and food addiction (i.e., both associations are mediated by weight-related self-stigma). Moreover, one study indicated that food addiction and psychological distress mediated the relationship between weight-related self-stigma and eating disorder [51], suggesting that the potential variables may exist in regulating the association between food addiction and eating behavior. In addition, some studies have indicated that food addiction may exacerbate weight-related stigma [16, 54], particularly weight-related self-stigma [54]. In other words, healthcare workers should be careful of using the term ‘food addiction’ when communicating with individuals who have weight problems. Individuals with obesity are likely to reinforce their weight-related self-stigma when they feel that they have the problems relating to food addiction [55].

Additionally, the association between psychological distress and food addiction has been widely reported [55]. Psychological distress is frequently reported as a consequence in response to weight-related stigma [5, 13, 35, 49, 50, 56]. However, other studies have focused on the mediating role of psychological distress in the development of eating disorders [47, 51]. Depression and anxiety, the two common types of psychological distress reported in the weight-related stigma mechanism, are highly associated with food addiction [57]. However, their roles in relation to regulating food addiction may be varied. The symptom of depression may derive from stress elicited by weight-related stigma and force individuals to adopt addictive food eating as an alleviation strategy [55]. However, other studies have shown that the intake of ultra-processed food may increase the likelihood of developing depression [58, 59]. However, anxiety has also been reported as a predictor of food addiction as well as being a risk factor for eating disorder and substance use in other addictive models (e.g., alcohol or drugs) [55]. In addition, anxiety predicts the consumption of sugar and saturated fat [60], which might share similar mechanisms of “comfort food” consumption under stressful conditions [61]. Therefore, the results of the present study supported the potential influences of psychological distress on food addiction and provided an insight into explaining its mediating effect.

The sequential mediated effect via weight-related self-stigma and psychological distress found in the present study enforces the association between perceived weight stigma and food addiction. There have been several previous studies investigating the potential mediators in the relationship between weight-related stigma and eating disorder. The results indicated that weight-related self-stigma is the most reported variable associated with eating disorder and can be mediated by psychological distress [51, 52]. Studies indicated the indirect effect of emotional dysregulation in the relationship between weight-related self-stigma and food addiction [52] and there is empirical evidence supporting that perceived weight stigma is also associated with eating disorder [35, 62, 63]. An additional mediating effect of eating disorder has been reported in the relationship between perceived weight stigma and psychological distress [35]. However, a result in the opposite direction regarding psychological distress in predicting food addiction has also been reported [55], suggesting that these relationships in explaining the mechanism underlying weight stigma may be bidirectional [5]. The present study found a sequential mediating effect of weight-related self-stigma and psychological distress in the relationship between perceived weight stigma and food addiction. This finding indicated that perceived weight stigma was associated with food addiction. The association was further mediated via weight-related self-stigma, followed by psychological distress, and their sequential effect appears to be important in the development of food addiction.

There are several limitations in the present study. First, the study population (i.e., Taiwanese university students) has relatively low levels of generalizability. The university students were relatively young and the present findings cannot necessarily be generalized to other age populations or cohorts, or to other populations on other countries and cultures. Second, the self-report measurements used in the present study may be subject to some study biases, such as recall bias (e.g., the weight-related stigma recalled by individuals may be affected by their current psychological condition) or social desirability bias (e.g., the participants in the present study may have deliberately reported they had less weight-related stigma). Third, a cross-sectional study design was adopted. Therefore, the results obtained from this type of design study are unable to determine any causality between the investigated variables. Given that the bidirectional associations may exist in the mechanisms of weight-related stigma, the cause-and-effect relationship may possibly be reversed or more be more complicated than expected. Fourth, a small number of university students may have difficulties in accessing Wi-Fi which means that the findings may not be generalizable to those without internet access.

Despite the limitations, the present study provides novel insights regarding the mechanism of weight-related stigma in relation to food addiction. To reduce stigmatization, stigma-reducing strategies such as psychoeducation or motivational interviewing [35] could be adopted. To improve weight status, weight management strategies such as mindful eating or keeping a food diary [55] could be adopted. In addition, to reduce the stigmatization derived from food addiction, a food addiction explanation model focusing on the etiology of food addiction could be adopted to help reduce its negative impact on weight-related stigma [64].

## Conclusions

The present study investigated the association between perceived weight stigma, weight-related self-stigma, and psychological distress with food addiction among Taiwanese university students. An additional mediated effect was examined. The results demonstrated significant associations between the investigated variables. An indirect sequential effect via weight-related self-stigma and psychological distress was found in explaining perceived weight stigma and food addiction. The findings of the present study provided novel insights regarding the mechanism of weight-related stigma in relation to food addiction and will help in the interventions that target the reduction of food addiction derived from weight-related stigma. Future studies should consider group analysis to consider the possible confounding factors such as gender or actual weight status, or the other targeted populations to provide more evidence in explaining weight-related stigma and food addiction.

## Data Availability

Data and code may be obtained from the corresponding author with reasonable request.

## References

[1] Wu YK, Berry DC (2018). Impact of weight stigma on physiological and psychological health outcomes for overweight and obese adults: a systematic review. J Adv Nurs.

[2] Tomiyama AJ, Epel ES, McClatchey TM, Poelke G, Kemeny ME, McCoy SK (2014). Associations of weight stigma with cortisol and oxidative stress independent of adiposity. Health Psychol.

[3] Sutin AR, Terracciano A (2013). Perceived weight discrimination and obesity. PLoS ONE.

[4] Tsenkova VK, Carr D, Schoeller DA, Ryff CD (2011). Perceived weight discrimination amplifies the link between central adiposity and nondiabetic glycemic control (HbA1c). Ann Behav Med.

[5] Hayward LE, Vartanian LR, Pinkus RT (2018). Weight stigma predicts poorer psychological well-being through internalized weight bias and maladaptive coping responses. Obesity.

[6] Lillis J, Levin ME, Hayes SC (2011). Exploring the relationship between body mass index and health-related quality of life: a pilot study of the impact of weight self-stigma and experiential avoidance. J Health Psychol.

[7] Tomiyama AJ. Weight stigma is stressful. A review of evidence for the cyclic obesity/weight-based stigma model. Appetite. 2014;82:8–15. 10.1016/j.appet.2014.06.108. 10.1016/j.appet.2014.06.108 24997407

[8] Ratcliffe D, Ellison N (2015). Obesity and internalized weight stigma: A formulation model for an emerging psychological problem. Behav Cogn Psychother.

[9] Griffin DW, Ross L. Subjective construal, social inference, and human misunderstanding. In: Advances in experimental social psychology(editor): pp. 319–59. Elsevier, 1991.

[10] Puhl RM, Brownell KD (2006). Confronting and coping with weight stigma: an investigation of overweight and obese adults. Obesity.

[11] Pearl RL, Himmelstein MS, Puhl RM, Wadden TA, Wojtanowski AC, Foster GD (2019). Weight bias internalization in a commercial weight management sample: prevalence and correlates. Obes Sci Pract.

[12] Torres SJ, Nowson CA (2007). Relationship between stress, eating behavior, and obesity. Nutrition.

[13] Hackman J, Maupin J, Brewis AA (2016). Weight-related stigma is a significant psychosocial stressor in developing countries: evidence from Guatemala. Soc Sci Med.

[14] Lazarus RS, Folkman S (1984). Stress, appraisal, and coping.

[15] Hebebrand J, Albayrak O, Adan R, Antel J, Dieguez C, de Jong J (2014). "Eating addiction", rather than "food addiction", better captures addictive-like eating behavior. Neurosci Biobehav Rev.

[16] Reid J, O’Brien KS, Puhl R, Hardman CA, Carter A (2018). Food addiction and its potential links with weight stigma. Curr Addict Rep.

[17] Gearhardt AN, White MA, Potenza MN (2011). Binge eating disorder and food addiction. Curr Drug Abuse Rev.

[18] Latner JD, Puhl RM, Murakami JM, O'Brien KS. Food addiction as a causal model of obesity. Effects on stigma, blame, and perceived psychopathology. Appetite. 2014;77:77–82. 10.1016/j.appet.2014.03.004. 10.1016/j.appet.2014.03.004 24630939

[19] Gearhardt AN, Hebebrand J (2021). The concept of "food addiction" helps inform the understanding of overeating and obesity: Debate Consensus. Am J Clin Nutr.

[20] Murphy CM, MacKillop J, Cottone P, Sabino V, Moore CF, Koob GF (2019). Food addiction and self-regulation. Compulsive eating behavior and food addiction.

[21] Gearhardt AN, White MA, Masheb RM, Morgan PT, Crosby RD, Grilo CM (2012). An examination of the food addiction construct in obese patients with binge eating disorder. Int J Eat Disord.

[22] Van Gaal LF, Mertens IL, De Block CE (2006). Mechanisms linking obesity with cardiovascular disease. Nature.

[23] Meadows A, Higgs S (2020). Internalized weight stigma and the progression of food addiction over time. Body Image.

[24] Berte DZ, Mahamid FA, Affouneh S (2021). Internet addiction and perceived self-efficacy among university students. I J Ment Health Addict.

[25] Vadeboncoeur C, Townsend N, Foster C (2015). A meta-analysis of weight gain in first year university students: Is freshman 15 a myth?. BMC Obes.

[26] Ferrari EP, Petroski EL, Silva DA (2013). Prevalence of body image dissatisfaction and associated factors among physical education students. Trends Psychiatry Psychother.

[27] Southerland J, Wang L, Richards K, Pack R, Slawson DL (2013). Misperceptions of overweight: associations of weight misperception with health-related quality of life among normal-weight college students. Public Health Rep.

[28] Tanenbaum HC, Felicitas JQ, Li Y, Tobias M, Chou CP, Palmer PH (2016). Overweight perception: associations with weight control goals, attempts, and practices among Chinese female college students. J Acad Nutr Diet.

[29] Lim H, Wang Y (2013). Body weight misperception patterns and their association with health-related factors among adolescents in South Korea. Obesity.

[30] Pakpour AH, Tsai MC, Lin YC, Strong C, Latner JD, Fung XCC (2019). Psychometric properties and measurement invariance of the Weight Self-Stigma Questionnaire and Weight Bias Internalization Scale in children and adolescents. Int J Clin Health Psychol.

[31] Essayli JH, Murakami JM, Wilson RE, Latner JD (2017). The impact of weight labels on body image, internalized weight stigma, affect, perceived health, and intended weight loss behaviors in normal-weight and overweight college women. Am J Health Promot.

[32] Alimoradi Z, Golboni F, Griffiths MD, Brostrom A, Lin CY, Pakpour AH (2020). Weight-related stigma and psychological distress: a systematic review and meta-analysis. Clin Nutr.

[33] Gan WY, Tung SEH, Kamolthip R, Ghavifekr S, Chirawat P, Nurmala I, et al. Evaluation of two weight stigma scales in Malaysian university students: weight self-stigma questionnaire and perceived weight stigma scale. Eat Weight Disord. 2022:1–10. 10.1007/s40519-022-01398-3. 10.1007/s40519-022-01398-3 35474190

[34] Nadhiroh SR, Nurmala I, Pramukti I, Tiara Tivany S, Tyas LW, Zari AP, et al. Weight Stigma in Indonesian Young Adults: Validating the Indonesian Versions of Weight Self-Stigma Questionnaire (WSSQ) and Percevied Weight Stigma Scale (PWSS). Asian J Soc Health Behav. In press.

[35] Lin CY, Strong C, Latner JD, Lin YC, Tsai MC, Cheung P (2020). Mediated effects of eating disturbances in the association of perceived weight stigma and emotional distress. Eat Weight Disord.

[36] Lillis J, Luoma JB, Levin ME, Hayes SC (2010). Measuring weight self-stigma: the weight self-stigma questionnaire. Obesity.

[37] Lin KP, Lee ML (2017). Validating a Chinese version of the Weight Self-stigma Questionnaire for use with obese adults. Int J Nurs Pract.

[38] Lovibond SH, Lovibond PF. Manual for the depression anxiety stress scales. Syndey, Australia: Psychology Foundation of Australia; 1996.

[39] Gong X, Xie X-y, Xu R, Luo Y-j. Psychometric properties of the Chinese versions of DASS-21 in Chinese college students. Chin J Clin Psychol. 2010;18(4):443–6. 10.16128/j.cnki.1005-3611.2010.04.020.

[40] Zanon C, Brenner RE, Baptista MN, Vogel DL, Rubin M, Al-Darmaki FR (2021). Examining the dimensionality, reliability, and invariance of the Depression, Anxiety, and Stress Scale–21 (DASS-21) across eight countries. Assessment.

[41] Gearhardt AN, Corbin WR, Brownell KD. Development of the Yale Food Addiction Scale Version 2.0. Psychol Addict Behav. 2016;30(1):113–21. 10.1037/adb0000136. 10.1037/adb0000136 26866783

[42] Penzenstadler L, Soares C, Karila L, Khazaal Y (2019). Systematic review of food addiction as measured with the Yale Food Addiction Scale: Implications for the food addiction construct. Curr Neuropharmacol.

[43] Chen I-H, Huang P-C, Lin Y-C, Gan WY, Fan C-W, Yang W-C, Tung SEH, Poon WC, Griffiths MD, Lin C-Y. The Yale Food Addiction Scale 2.0 and the modified Yale Food Addiction Scale 2.0 in Taiwan: Factor structure and concurrent validity. Front Psychiatry. 2022;13:1014447. 10.3389/fpsyt.2022.1014447. 10.3389/fpsyt.2022.1014447 PMC9732099 36506452

[44] Zhang H, Tong T, Gao Y, Liang C, Yu H, Li S, et al. Translation of the Chinese version of the modified Yale Food Addiction Scale 2.0 and its validation among college students. J Eat Disord. 2021;9(1):116. 10.1186/s40337-021-00471-z. 10.1186/s40337-021-00471-z PMC8444594 34530921

[45] Hayes AF (2017). Introduction to mediation, moderation, and conditional process analysis: a regression-based approach.

[46] Lin CY, Tsai MC (2016). Effects of family context on adolescents’ psychological problems: moderated by pubertal timing, and mediated by self-esteem and interpersonal relationships. Appl Res Qual Life.

[47] O'Brien KS, Latner JD, Puhl RM, Vartanian LR, Giles C, Griva K (2016). The relationship between weight stigma and eating behavior is explained by weight bias internalization and psychological distress. Appetite.

[48] Fan CW, Liu CH, Huang HH, Lin CY, Pakpour AH (2021). Weight stigma model on quality of life among children in Hong Kong: a cross-sectional modeling study. Front Psychol.

[49] Lin CY, Tsai MC, Liu CH, Lin YC, Hsieh YP, Strong C (2019). Psychological pathway from obesity-related stigma to depression via internalized stigma and self-esteem among adolescents in Taiwan. Int J Environ Res Public Health.

[50] Chan KL, Lee CSC, Cheng CM, Hui LY, So WT, Yu TS (2019). Investigating the relationship between weight-related self-stigma and mental health for overweight/obese children in Hong Kong. J Nerv Ment Dis.

[51] Ahorsu DK, Lin CY, Imani V, Griffiths MD, Su JA, Latner JD (2020). A prospective study on the link between weight-related self-stigma and binge eating: role of food addiction and psychological distress. Int J Eat Disord.

[52] Baldofski S, Rudolph A, Tigges W, Herbig B, Jurowich C, Kaiser S (2016). Weight bias internalization, emotion dysregulation, and non-normative eating behaviors in prebariatric patients. Int J Eat Disord.

[53] Vartanian LR, Porter AM (2016). Weight stigma and eating behavior: a review of the literature. Appetite.

[54] Parnarouskis L, Jouppi RJ, Cummings JR, Gearhardt AN (2021). A randomized study of effects of obesity framing on weight stigma. Obesity.

[55] Wiss D, Brewerton T (2020). Separating the signal from the noise: How psychiatric diagnoses can help discern food addiction from dietary restraint. Nutrients.

[56] Puhl RM, Heuer CA (2009). The stigma of obesity: a review and update. Obesity.

[57] Burrows T, Kay-Lambkin F, Pursey K, Skinner J, Dayas C (2018). Food addiction and associations with mental health symptoms: a systematic review with meta-analysis. J Hum Nutr Diet.

[58] Gomez-Donoso C, Sanchez-Villegas A, Martinez-Gonzalez MA, Gea A, Mendonca RD, Lahortiga-Ramos F (2020). Ultra-processed food consumption and the incidence of depression in a Mediterranean cohort: the SUN Project. Eur J Nutr.

[59] Adjibade M, Julia C, Alles B, Touvier M, Lemogne C, Srour B (2019). Prospective association between ultra-processed food consumption and incident depressive symptoms in the French NutriNet-Sante cohort. BMC Med.

[60] Fonseca N, Molle RD, Costa MA, Goncalves FG, Silva AC, Rodrigues Y (2020). Impulsivity influences food intake in women with generalized anxiety disorder. Braz J Psychiatry.

[61] Tomiyama AJ, Dallman MF, Epel ES (2011). Comfort food is comforting to those most stressed: evidence of the chronic stress response network in high stress women. Psychoneuroendocrinology.

[62] Durso LE, Latner JD, Hayashi K (2012). Perceived discrimination is associated with binge eating in a community sample of non-overweight, overweight, and obese adults. Obes Facts.

[63] Linde JA, Wall MM, Haines J, Neumark-Sztainer D (2009). Predictors of initiation and persistence of unhealthy weight control behaviours in adolescents. Int J Behav Nutr Phys Act.

[64] O'Brien KS, Puhl RM, Latner JD, Lynott D, Reid JD, Vakhitova Z (2020). The effect of a food addiction explanation model for weight control and obesity on weight stigma. Nutrients.

